# Developing a complex intervention to support pregnant women with mild to moderate anxiety: application of the Medical Research Council framework

**DOI:** 10.1186/s12884-020-03469-8

**Published:** 2020-12-14

**Authors:** Kerry Evans, Helen Spiby, C. Jane Morrell

**Affiliations:** 1 University of Nottingham, 12th Floor Tower Building, Nottingham, NG7 2RD UK; 2 School of Nursing and Midwifery, University of Queensland, Brisbane, Australia

## Abstract

**Background:**

To design and develop an intervention to support women with symptoms of mild to moderate anxiety in pregnancy.

**Methods:**

The development followed the MRC framework for complex interventions, utilising psychological theory, review level evidence and professional and public involvement. Two systematic reviews were completed which helped identify potentially beneficial intervention components. The theory underpinning the components was explored to consider the potential benefit for women with mild to moderate anxiety symptoms in pregnancy. Methods of delivering the intervention within maternity services were explored. The intervention comprised: group discussions, one to one support and assisted self-help resources. Midwives were identified as ideally placed to facilitate the intervention supported by midwifery support workers. A bespoke training package was provided by subject experts to prepare the facilitators.

**Results:**

The absence of established interventions and a paucity of evidence based approaches for pregnant women with symptoms of mild to moderate anxiety indicated the need for a rigorous and systematic approach to the intervention design. This approach led to the development of an intervention feasible for implementation in maternity care systems tailored to the needs of pregnant women. The involvement of a multi-professional advisory team and active engagement of service users helped to consider the acceptability of the intervention for women and the feasibility of delivering the intervention in the context of maternity care.

**Conclusion:**

The MRC Framework provided useful overarching guidance to develop a midwife facilitated intervention for women with symptoms of anxiety in pregnancy. The framework assisted the development of a robust rationale for each intervention component and considered the processes of evaluation and implementation into maternity care systems.

## Background

Anxiety disorders are reported as the sixth leading cause of disability globally, with women accounting for 65% of disability adjusted life years. Costs of additional use of public services, productivity losses and quality adjusted life year lost for women with anxiety in the perinatal period and continuing up to 10 years after birth were estimated at £35,000 for the mother and child [9]. Symptoms of anxiety are experienced by many pregnant women; prevalence of antenatal anxiety disorders has been reported between 13 and 15% in the UK and US [63]. There were 657,076 live births in England and Wales in 2018 and it is therefore likely that around 90,000 women experience symptoms of anxiety in pregnancy each year.

Anxiety disorders in pregnancy usually present with similar symptoms to anxiety disorders at other times [63]. However, concerns about pregnancy may present as the predominant feature [10]. Although mild anxiety in pregnancy is a normal adaptive process, symptoms become problematic when they consume a large proportion of a woman’s time, when a woman is unable to focus on other tasks and when symptoms interfere with everyday life Wenzel [81]. Anxiety disorders can result in significant disability for sufferers and possible negative effects on the fetus [63]. Elevated and prolonged anxiety has been associated with pre-term birth, fetal growth restriction and behavioural problems in developing children [28, 33, 50]. Antenatal anxiety has been reported to have a negative impact on women’s confidence in mothering, satisfaction with their infants and predict post-traumatic stress disorder and postnatal depression [36, 38, 42].

In the UK, midwives provide care for every pregnant woman and are ideally placed to identify mental health concerns and support emotional wellbeing [53]. Maternity care previously focused on physical wellbeing; greater support for the major psychological transition women experience in pregnancy and motherhood is required [1]. Psychological interventions may be beneficial in reducing symptoms of anxiety but need to be evaluated in pregnant populations to strengthen the evidence base.

The aim of interventions is to provide suitable, timely support and treatment to prevent an escalation of symptoms and improve women’s ability to cope [53]. Perinatal mental health is a priority area identified in the National Health Service long term plan [2] which aims to provide an additional 24,000 women each year with access to specialist perinatal mental healthcare. Priority areas include increasing access to evidence-based care including psychological therapies and mental health assessment. All women identified with mild to moderate anxiety should be offered a range of support tailored to their needs [26]. However, services to support women’s mental health are not always readily available and need to be strengthened [53]. Many women stop taking anxiety medication in pregnancy, due to uncertainty surrounding the risk of teratogenicity [6] and non-pharmacological interventions are recommended as the initial treatment option [63]. There are no existing systematic reviews which evaluate interventions to improve mild to moderate anxiety in pregnancy. New interventions need to be developed in response to the theoretical and evidence base (Medical Research Council, MRC, [24]).

This paper reports the stages of an intervention development utilising the MRC framework for developing complex interventions [24]. The aim of the intervention was to support women with mild-moderate symptoms of anxiety in pregnancy.

## Methods

**Fig. 1:**
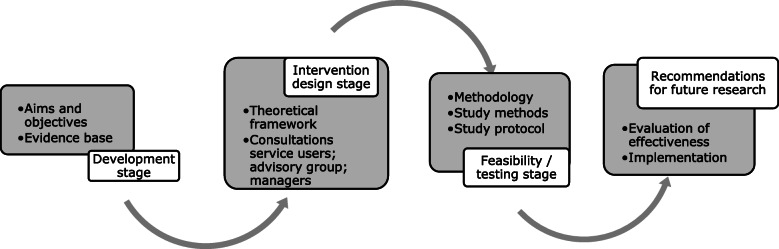
Key elements of the development and evaluation process (Based on MRC, [24])

The MRC described complex interventions as: 1) including several interacting components; 2) sensitive to the context in which they are delivered; 3) having a causal chain linking the intervention to outcomes; 4) having a range of possible outcomes [24]. It was considered that a new intervention would need to operate within different maternity settings and be delivered to different populations of pregnant women. The choice of intervention components should include consideration of how the mechanisms of change would function within the context of maternity care structures and propose ways the mechanisms would influence women’s symptoms of anxiety. Therefore, the intervention was considered as ‘complex’ and the stages of the intervention development followed the general principles outlined by the MRC theoretical and modelling phases for complex interventions [24] (Fig. 1).

### Findings

#### Identifying the evidence base

The MRC state that the development of a complex intervention should begin by identifying the relevant, existing evidence base [24]. Existing reviews which have evaluated the effectiveness of interventions on anxiety outcomes in pregnancy have focused on depression, mind-body or pharmacological interventions or included women with severe anxiety. Therefore two systematic reviews were completed to identify the evidence base for non-pharmacological interventions delivered to women with mild to moderate anxiety in pregnancy [30, 31]. The systematic reviews asked the following questions:

- How effective are non-pharmacological interventions in reducing the symptoms of mild to moderate anxiety in pregnancy?
- How acceptable and beneficial are non-pharmacological interventions for reducing the symptoms of mild to moderate anxiety?

The two systematic reviews concluded that interventions, specifically designed to support pregnant women with mild to moderate anxiety have mainly been evaluated in small scale studies. Studies evaluated different intervention designs for different populations and overall results were inconclusive regarding intervention effectiveness. Although no particular design which could be directly recommended for clinical practice was identified, the synthesised review findings helped identify components likely to increase the effectiveness and acceptability of the intervention.

There was some evidence of benefit for group interventions, women valued the opportunity to share experiences, reducing feelings of isolation, and accessing group support [8, 12, 17, 29, 32, 37, 39, 72, 79, 85]. Some women felt they benefitted from having an individual discussion with their healthcare professionals (HCP) [19, 23]. Women were motivated to self-select into intervention studies however, some had concerns about disclosing anxiety symptoms and joining groups. There was some evidence of benefit for multi-session interventions, and women identified group sessions as helpful once groups became established. Studies which reported an improvement in anxiety scores included group mindfulness [39], mindfulness based cognitive therapy [32], motivational interviewing [12], relaxation [8, 21, 77, 78] or CBT interventions [54]. Women welcomed interventions which presented options for managing their symptoms and included peer or professional support [31].

#### Identifying appropriate theory

The theory underpinning the potentially beneficial intervention components as identified in the two reviews were explored (Table 1). This process strengthened the rationale for the final intervention design and helped to define the process of change in relation to anxiety symptoms in pregnancy [57]. The development of complex interventions requires researchers to develop an awareness of the relevant theory underpinning intervention components to increase the likelihood of the effectiveness of the intervention design [24, 34]. A description of the intervention’s underlying theoretical basis should include specific theories, theoretical positions, and frameworks as well as empirical evidence which may have been conducted in different settings or countries [56].

**Table 1:** Summary of the findings from the systematic reviews and the theory underpinning the intervention components

	Women’s views on intervention components	Theory
Group and individual interventions		
Interventions delivered to groups of pregnant women	• Able to share experiences • Accessed group support • Reduced feelings of isolation • Helped to normalise women’s experiences	• Social support °Experiential knowledge °Social learning °Social comparison °Peer support
Interventions delivered to individuals	• Received support from HCPs • Provided reassurance and guidance	• Therapeutic relationships °Collaborative role theory °Relational continuity °Social influence
Intervention components		
Mind-body	• Provided options and coping strategies for managing anxiety symptoms • Learned breathing and relaxation techniques • Learned to recognise and adapt to anxious thoughts • Felt more positive about the future	• Awareness, self-regulation and adapted behaviour • Relaxation response
Psychological	• Developed an understanding of the causes of anxiety in their lives and self-awareness of their thought patterns. • Helped women respond in a more positive way to situations and feelings, before negative thought patterns could escalate.	• Cognitive behavioural mechanisms

### Social support theory

Social support may have a positive effect on wellbeing, such as providing: 1. compassion, reassurance and a sense of self-worth; 2. access to new contacts and information to help develop problem solving skills; 3. reducing feelings of uncertainty and develop a sense of control; 4. providing instrumental support to reduce the frequency and duration of stressors; and 5. influencing positive health behaviours [40]. Social support pathways include components of experiential knowledge; social learning theory; social comparison theory and the helper-therapy principle [70]. Individuals resolve their problems through sharing their experiences of mental illness with others who are experiencing similar situations [14] and can benefit by learning from others who have succeeded in managing their symptoms [75].

### Therapeutic relation theory

Collaborative therapeutic relationships enable pregnant women to feel physically and psychologically supported which facilitates confidence building and self-efficacy [20]. Continuity of carer from a midwife known to the woman throughout pregnancy and the intrapartum period has been associated with improved health outcomes for women and babies [71]. Benefits include an increased sense of trust, choice and control. Social influence theory recognises that the HCP’s may be seen as a source of social power due to their access to information, resources and services. While this may be beneficial, it is also associated with negative outcomes if individuals are influenced or coerced into compliance to gain access to services or information. Excessive information seeking and reassurance seeking are common features of anxiety disorders and can have a negative impact on outcomes and the practitioner–service user relationship [68]. A pregnant woman with health anxiety may continually or excessively seek reassurance about fetal growth, the progress of their pregnancy and about the birth [10]. HCPs need to be aware of possible service user motivations for seeking reassurance about their health and wellbeing and suggest strategies, such as CBT, to help modify negative behavioural patterns [83].

### Mind-body approaches

Awareness of mind and body experiences enables an individual to direct their attention to their breathing or another object of focus, to prevent elaborative ruminative thought processing [35, 66]. Mind-body interventions like yoga, guided imagery, mindfulness or hypnotherapy may be effective for reducing anxiety as they are thought to induce mental relaxation and alter negative thinking related to anxiety ([52]. Mind-body approaches are intended to modify an individual’s perceptions of stressful events which can lead to improvements in adapted behaviour and develop coping strategies [52]. The relaxation response is thought to counteract the stress response of anxiety. Physiological mechanisms and adjustments are activated when an individual engages in repetitive mental or physical activity and is able to passively ignore anxious thoughts [51].

### Cognitive-behavioural mechanisms

In the treatment of anxiety disorders, the aim of cognitive behavioural therapy (CBT) is to reduce anxious feelings by undoing prior learning or by providing new, more adaptive learning experiences and changing cognitive and behavioural responses to anxiety [84]. Increasing an individual’s awareness of unwanted emotions and behaviours is thought to generate a number of alternative responses. This helps the individual to decide on a course of action and monitor the outcome to re-enforce positive coping strategies [16]. CBT for anxiety disorders may include components of:

- Psycho-education on the nature of fear/anxiety.
- Cognitive restructuring to challenge the truth of anxious thoughts and develop alternative thoughts to better reflect their experience [16].

Behavioural exposure components of CBT require further consideration in the context of pregnancy. There are very few studies evaluating exposure-based CBT due to concerns around potential harm to the fetus [4, 48].

### Multi-component approach

Many of the interventions identified in the systematic reviews had multiple components: psycho-education; relaxation; peer support; and professional support. This multi-component approach was reflected in the interconnected theoretical approaches which underpinned existing intervention components. For example, CBT techniques are often incorporated within therapeutic relationship approaches and can be accessed as a resource within peer support models.

**Fig. 2:**
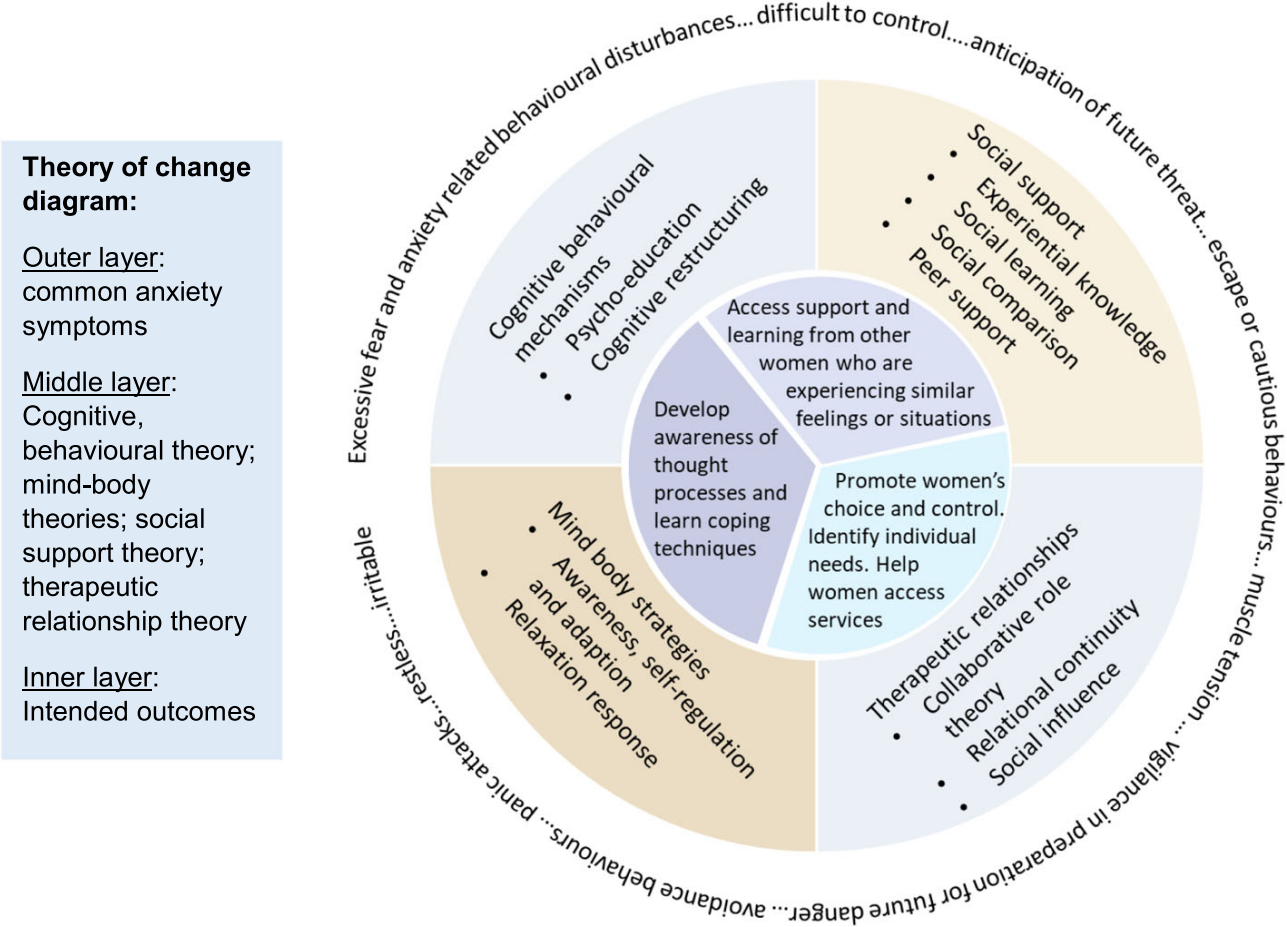
Theoretical model outlining the mechanisms which are considered to result in an improvement in anxiety symptoms for pregnant women

A theoretical model was developed to map the potential mechanisms and their usefulness in meeting the needs of pregnant women with symptoms of mild to moderate anxiety (Fig. 2). Exploring the theoretical base highlighted that positive change can occur though: 1. developing collaborative relationships with women which aim to promote women’s choice and control over their care. 2. receiving support from HCPs who both understand women’s individual needs and can also help them access services; 3. accessing support and learning from other women who have experienced / are experiencing similar feelings or situations; 4. developing strategies to help women develop an awareness of their thought processes and learn techniques to improve the way they cope with anxiety. Mind-body and/or CBT approaches were considered as appropriate components of the intervention design.

### Additional considerations and motivations informing the intervention design

**Fig. 3:**
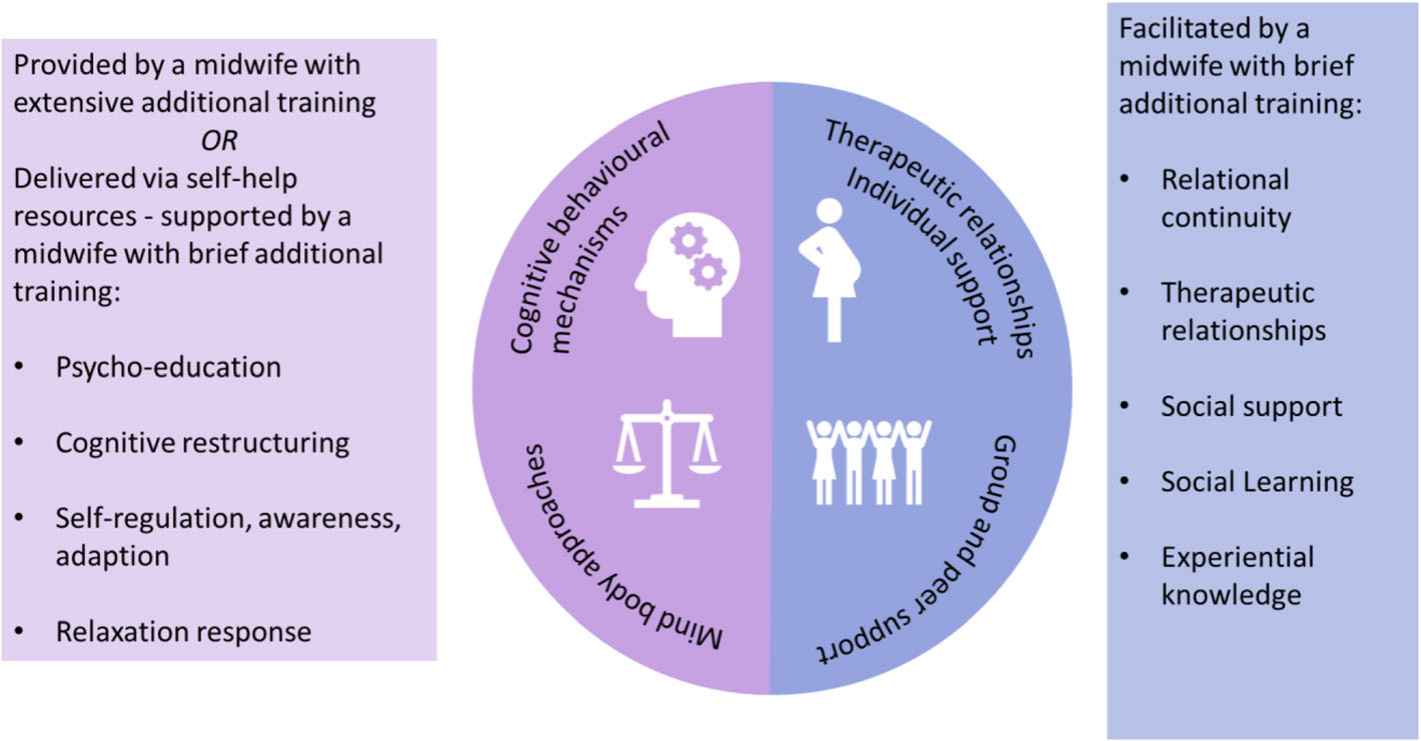
Methods of delivery for the intervention components

In response to the increased focus on the role of the midwife to support the psychological and emotional wellbeing of women in pregnancy [53], the development work explored ways in which women could be supported by midwives within midwives’ current scope of practice [67]. It was considered that a midwife could facilitate peer groups, acting as a resource to the women. Midwife facilitation may be more appropriate when groups are establishing, however the role of the professional in peer groups should not interfere with the potential benefits derived when group members help others in the group [18]. In maternity care, the role of the HCP in breastfeeding support groups has been reported to “normalise or counteract extreme views and help women to distinguish between fact, anecdote and myth” ([41], page 143). In a group based antenatal care study (Andersson et al.[3], women welcomed midwives who were less structured in their approach to group facilitation. They appreciated midwives contributing their expertise in antenatal care and helping to address topics women found difficult to introduce. To maximise the benefit of social learning mechanisms, women may benefit from hearing the experiences of other women who have been through similar experiences who can share their success stories and inspire hope [25, 55, 69]. Women who feel isolated in pregnancy or have poor social support may benefit from peer group approaches, however some women may not feel confident to share their situations or feelings within a group. Women may have additional pregnancy related or mental health concerns which they would prefer to discuss individually with a midwife who can provide maternity expertise and support referrals or signposting to other specialist services such as Increasing Access to Psychological Therapies (IAPT). The options for the delivery of the intervention components, considering the feasibility of employing midwife facilitators and facilitator training requirements were mapped (Fig. 3). The advisory group raise concerns that the training to deliver CBT and mindfulness-based interventions was intensive, with training usually taking 1 year or more to complete. Also, at the time of the study development readily accessible training courses were not focused on delivering interventions to pregnant women. Recent studies have highlighted the effectiveness of interpersonal psychotherapy and CBT interventions to prevent postnatal depression which can be delivered by nurses, midwives and health visitors in antenatal care settings and require brief initial training [45, 46], and a brief midwife-led CBT intervention for maternal anxiety is in progress [82]. For this intervention, it was considered that the therapeutic intervention components (mind body and cognitive behavioural approaches) could be delivered through supported use of self-help resources. Guided self-help has been reported as an effective intervention for depression and anxiety in general populations [73] and has been used as a stand-alone intervention or alongside group interventions for pregnant women with anxiety, stress and depression [30]. Potential self-help resources were identified evaluated using IAPT criteria [43].

#### Modelling process and outcomes

For this study, potential intervention components and processes were tested through consultations with a study advisory group and a maternity research public involvement group. The advisory group consisted of the head of nursing and midwifery research at the local NHS trust, a community psychiatric nurse, a midwife manager, a service user, consultant clinical psychologist and mental health training providers. Service users provided insight into how the intervention would be accessed and used and ensured the intervention was relevant to the needs of pregnant women [44, 58]. Both groups supported the proposed intervention components and helped to identify methods of delivery for the intervention which considered: the context and methods for introducing the intervention, assessing eligibility, method of delivery and facilitation of peer groups; and delivery of the therapeutic components. Rather than having two midwife facilitators, service managers identified that a midwifery support worker (MSW) could provide support to the midwife during the groups and co-facilitate the intervention. A bespoke training framework was developed for midwives and MSWs which referred to existing perinatal competency frameworks [64, 65]. Experienced mental health training providers developed a three day training workshop which included a range of educational and learning approaches e.g. role play, lectures and the completion of an information and reflective workbook.

### Intervention protocol

**Fig. 4:**
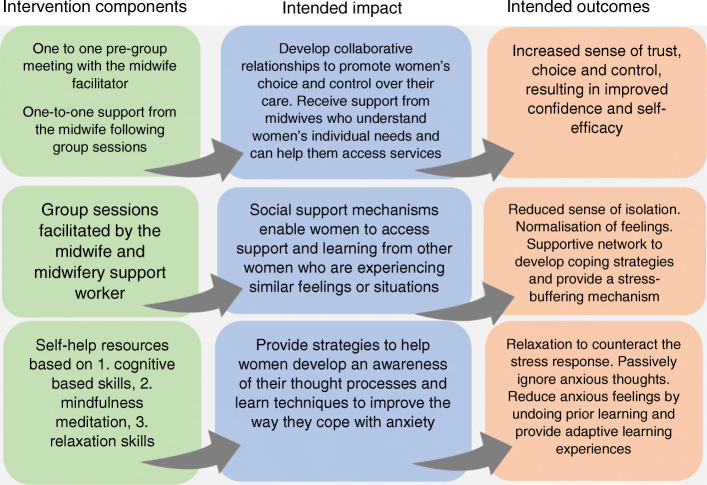
Intervention components, intended impact and outcomes

Following the evaluation of the evidence base, exploring the theoretical base and consultations with stakeholder groups, a protocol was developed for the intervention ([56], Fig. 4, Tables 2 and 3).

**Table 2:** Foundation and rationale for the final intervention design

	Description	Foundation and rationale
**Sample population**	Nulliparous women in the second trimester of pregnancy.	**Advisory group and service user group:** focus on nulliparous women forpreliminary testing (facilitate data analysis and more likely to have and abilityto participate).
**Participant eligibility**	**Inclusion criteria:** 1. Nulliparous pregnant women 2. Self- report mild-moderate anxiety **Exclusion criteria:** 1.Receiving treatment for a severe and enduring mentalhealth condition. 2. Complex social factors (NICE [60]).	**Current clinical policy:** women with severe mental health concerns andcomplex social factors have established referral pathways to specialist services. **Eligibility screening method:** Consider using validated anxiety measurementtools (NICE [61], Sinesi et al. [76], Nath et al. [59]).
**Inclusion screening**	The anxiety measurement tool will be administered by thecommunity midwife to indicate women who meet the cut-offscore for mild to moderate.	**Systematic review:** rationale for inclusion screening should be discussedwithin a supportive context. **Advisory group suggested:** midwives may require training of anxiety tooladministration. **Service user feedback:** inclusion screening would be acceptable; the midwifeshould be aware of concerns women may have about disclosing symptoms.
**Intervention facilitator**	The intervention will be facilitated by midwives and co-facilitatedby MSWs. They will receive training to deliver the intervention.One midwife and one support worker will facilitate each group.	**Systematic review:** delivered by psychiatrists, psychologists, midwives,instructors, self-help and volunteers. **Advisory group suggested:** women may be more willing to seek supportfrom midwives than mental health professionals. **Service user feedback:** supported midwife facilitation **Consultations with trainers**: two facilitators optimal for group interventions. **Service Manager feedback:** Suggestions to include support workers asco-facilitators.
**Intervention components**	Delivered in three components: **Component 1:** one to one pre-group meeting with the midwifefacilitator.	**Systematic review:** some women had concerns about disclosing symptomsand feared the judgment of others (in groups). Initial meetings with facilitatorshelped women feel more confident to join the group. **Advisory group:** one to one meetings provide opportunity to discussconcerns and answer questions.
	**Component 2:** Four sessions facilitated by a midwife and MSW.Sessions will take place fortnightly and will be held in communityhealthcare centres. Each session will last for 90 min (either earlyevening or weekends).	**Systematic review:** group discussion sessions were highlighted as animportant and valued component **Advisory group:** self-help resources with discussion sessions supported asan option. CBT may not be feasible for the study due to the intensive trainingrequired for delivery. **Advisory group:** support for community locations **Service user feedback:** groups may help normalise experiences and buildsocial support. **Service user feedback:** offer outside daytime working hours.
	**Component 3**: Choice of self-help resources for completionbetween sessions:	**Systematic review:** some participants reported self-help interventions aschallenging but also helpful **Advisory group:** self-help resources supported as an option **Service user feedback:** considered useful, women should be able to choosefrom different formats.

**Table 3:** Key assumptions, process interventions and indicators relating to the Theory of Change for the proposed intervention

Assumptions a	1. Midwives and midwifery support workers are motivated to apply to be trained and participate as intervention facilitators; Maternity mangers are willing to release midwives and midwifery supporters time to complete training and facilitate the intervention; Intervention facilitators are supported by specialist PMH teams and professional midwifery advocates
	2. Community midwives are confident and competent to delivery anxiety screening tools; Community midwives feel confident to discuss perinatal mental health with women and create the right context for women to disclose their symptoms and access supportive services
	3. Specialist perinatal mental health teams and psychological services support the intervention as a service for women with sub-threshold symptoms of anxiety Specialist perinatal mental health teams and psychological services are willing to support intervention facilitators by providing training in supporting women with anxiety and provide advice and referral pathways for women who are identified as having more severe symptoms or requiring more specialist support
	4. Women are willing to disclose their symptoms and women with mild to moderate symptoms of anxiety are willing to attend and engage with the intervention Women who develop more severe symptoms or are identified by intervention facilitators are requiring specialist support are willing to be accept a referral to specialist PMH services for assessment and treatment
Interventions iv	2. Recruitment and training of facilitators
	1. Intervention co-ordinator trained to monitor the intervention fidelity, measure outcomes and support facilitators across maternity systems
	3. Training of community midwives to effectively screen for symptoms of anxiety and refer women with mild to moderate anxiety to intervention facilitators Intervention facilitators to raise awareness of the intervention in local community teams
	4. Establish a multi-disciplinary stakeholder team to support the implementation of the intervention
	5. Women who develop more severe symptoms or are identified by intervention facilitators are requiring specialist support are referred to specialist PMH services for assessment and treatment
Indicators id	2. Facilitators assessment of the usefulness of training and preparedness to facilitate the intervention
	3. 80% of community midwives are aware of the intervention and know how to refer women to intervention facilitators; 80% of women who are identified with mild to moderate symptoms of anxiety and are eligible for participation are referred to intervention facilitators
	1. Intervention fidelity assessment reaches pre-agreed standards; Facilitators feel well supported in their roles; The intervention is implemented across maternity care systems
	4. Women attend 75% of intervention sessions; Rates of appropriate referrals to specialist services
	5. Women report an improvement in generalised and pregnancy-specific anxiety scores (pre-agreed % in improvement); Women’s evaluation of the acceptability and usefulness of the intervention; Improvement in infant outcomes; Improvement in perinatal mental health in the postnatal period (3, 6 and 12 months)

**Fig. 5:**
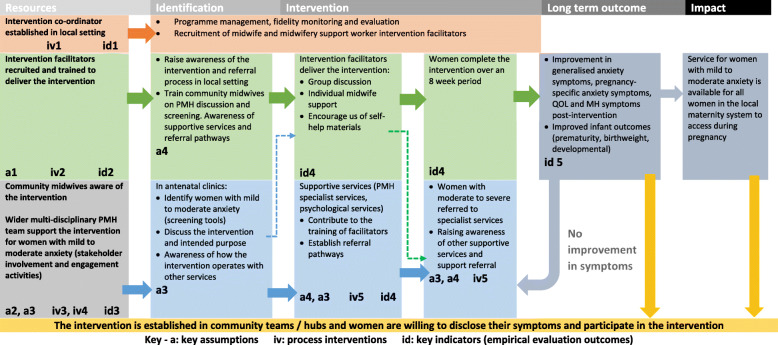
Theory of Change Map for an intervention to support women with symptoms of mild to moderate anxiety in pregnancy

The MRC [24] state that the future implementation of the intervention needs to be considered at an early stage of development. This should ask questions about whether implementation would be possible, who the key stakeholders are and what information they may need to implement changes in practice. De Silva et al. [27] proposed that the current MRC guidance could be strengthened by incorporating Theory of Change (TOC) into the design and evaluation of complex interventions To help identify the intervention processes and success indicators a TOC map was developed (Fig. 5). TOC defines how and why an initiative works, providing a pragmatic framework to describe how the intervention affects change [27, 80]. Each pre-condition for the intervention is evidence based and measured through an indicator. The TOC can help reduce future implementation failures as weak links in the causal pathway can be tested, revised and strengthened. The TOC map set out to answer a series of questions which asked how the intervention could be integrated into routine practice and identifying how the intervention could be empirically tested in future definitive research [13, 27].

## Discussion

The adoption of the MRC framework provided useful guidance to inform the development of a novel evidence-based intervention underpinned by the theoretical base to improve symptoms of mild to moderate anxiety in pregnant women. The theory and evidence base were synthesised to identify potential intervention components. The modelling phase clarified the intended impact and outcomes of the intervention components and methods of delivery.

Although non-pharmacological interventions are recommended as the initial treatment option, psychological interventions developed specifically for pregnant women with mental health concerns have demonstrated promising results but have not been rigorously evaluated in large studies. Furthermore the theoretical base to improve symptoms of anxiety has not been developed specifically for a pregnant population [30, 47]. The MRC framework [24] was used as the overarching guidance to assist the development of a psycho-social intervention for pregnant women. The framework was particularly useful in clarifying the intervention components, linking the evidence base and theory with the intended outcomes to provide a robust rationale for each component and defining the mechanisms of change. Questions regarding the eventual implementation of the intervention were addressed through application of the MRC guidance and mapping the TOC, helping to consider the intervention and study processes and highlighted the value of stakeholder engagement to increase the intervention feasibility and acceptability. For the proposed intervention, the TOC was developed in collaboration with stakeholders and the study advisory group, informed from the evidence base and the views of women and healthcare professionals working in perinatal mental health or maternity care. This enabled key assumptions and barriers to be identified and define the methods of measurement for patient-level and service level factors, for example:

- Facilitator training (uptake and participants’ evaluation)
- Acceptability and uptake (uptake and attendance rates for each intervention component across the various care settings and service user groups)
- Integration in maternity care systems and additional supportive services (intervention fidelity, referral rates in perinatal mental health services, time taken for screening, delivery and facilitation)

Levati et al. [49] conducted a scoping review of strategies used optimise complex interventions prior to definitive testing. A range of different guidelines were employed by authors in the development stages, with the MRC framework used in 17 of the 27 included studies with a few studies adopting the mapping framework [7] and the MOST framework [22]. The current MRC framework [24], while stressing the importance of context, lacked specific guidance on methods to define and describe the context of the intervention. It was important for the proposed intervention that, in addition to the local maternity care structures, wider policy recommendations for the intervention development were defined and considered. This was particularly relevant as the intervention development was being conducted during the publication of new national maternity care policy and would need to be operational in both existing and future maternity care contexts (National Maternity [62]). In addition, the involvement of midwives to facilitate the intervention was motivated from the wider midwifery care literature which stressed the need to strengthen the role of the midwife in promoting women’s mental and emotional wellbeing. Thus, developing an intervention which could be delivered within midwives’ scope of practice, with minimal additional resources and which could be integrated into midwifery services was of particular importance. Bleijenberg et al. [11] identify that the ways the context interact with the intervention are not always addressed by existing intervention development guidance. Information regarding the implementation context, the recipients, and the providers can help optimise the ability to operationalise the intervention before proceeding to the next phase of evaluation. Our experience with developing the proposed intervention supports the recommendations by Bleijenberg et al. [11] that additional elements are incorporated into the MRC Framework development phase, particularly problem identification and definition; determination of recipients’ and providers’ needs; and examination of current practice and context. Such information will assist future evaluations of the intervention and consider the relevance for other populations or settings.

In addition to the MRC framework, the CReDECI 2 reporting guidance for comprehensive reporting of the development, piloting, and evaluation of complex interventions in healthcare (EQUATOR network, [56], provided further useful considerations. When reporting methodological aspects of future evaluations, information about intervention modelling should be clearly defined. This should include the target setting, macro level conditions (i.e. legal and political aspects of midwifery scope of practice, education of midwives and support workers), the meso level (i.e. system level maternity networks, supportive services) and the micro level (i.e. midwifery care team composition and caseload). Further development is required to ensure the cultural appropriateness of the intervention and identify ways the intervention can be adapted to meet the needs of women with complex social factors. Effective recruitment strategies need to be developed to address potential disparity of the intervention within current maternity care structures. Service users, local healthcare and community groups should be involved in designing the protocol and materials for cultural relevancy and in promoting the study in different communities [15, 24, 74]. Atif et al. [5] developed an antenatal psychological intervention for women in Pakistan following the MRC framework. The authors conducted qualitative research with women and care providers to consider the needs of the target population based on consideration of their problems, demographic and contextual factors. The findings were combined with the established theoretical and evidence-based approaches to inform and adapt the intervention design.

## Conclusions

The MRC Framework provided useful overarching guidance to develop a midwife facilitated intervention for women with symptoms of anxiety in pregnancy. The framework enabled a thorough consideration of the theoretical and evidence base and highlighted the importance of stakeholder engagement to model the intervention processes. This resulted in clear rationale for the intervention components and considered the processes of evaluation and implementation into maternity care systems. The intervention development was strengthened by mapping the theory of change for implementation which considered the local context, maternity care processes and empirical performance indicators. Inclusion of these additional processes in addition to the MRC recommendations may assist future researchers with an interest in developing the evidence-base for women with anxiety in pregnancy and facilitate the evaluation, adaption, and development of interventions.

## Data Availability

Not applicable, no primary data was collected.

## Abbreviations

HCP: Healthcare Professional
IAPT: Increasing Access to Psychological Therapies
MRC: Medical Research Council
MMHA: Maternal Mental Health Alliance
MSW: Midwifery Support Worker
NIHR: National Institute for Health and Care Excellence
NHS: National Health Service
TOC: Theory of Change
